# Volatile Profile of Mead Fermenting Blossom Honey and Honeydew Honey with or without *Ribes nigrum*

**DOI:** 10.3390/molecules25081818

**Published:** 2020-04-15

**Authors:** Giulia Chitarrini, Luca Debiasi, Mary Stuffer, Eva Ueberegger, Egon Zehetner, Henry Jaeger, Peter Robatscher, Lorenza Conterno

**Affiliations:** 1 Laimburg Research Centre, Ora (BZ), 39040 Auer, Italy; giulia.chitarrini@laimburg.it (G.C.); luca.debiasi@laimburg.it (L.D.); mary.stuffer@students.boku.ac.at (M.S.); eva.ueberegger@laimburg.it (E.U.); peter.robatscher@laimburg.it (P.R.); 2 Institute of Food Technology, University of Natural Resources and Life Sciences (BOKU), 1190 Vienna, Austria; egon.zehetner@boku.ac.at (E.Z.); henry.jaeger@boku.ac.at (H.J.)

**Keywords:** gas chromatography-mass spectrometry, fermentation, honey, black currant

## Abstract

Mead is a not very diffused alcoholic beverage and is obtained by fermentation of honey and water. Despite its very long tradition, little information is available on the relation between the ingredient used during fermentation and the aromatic characteristics of the fermented beverage outcome. In order to provide further information, multi-floral blossom honey and a forest honeydew honey with and without the addition of black currant during fermentation were used to prepare four different honey wines to be compared for their volatile organic compound content. Fermentation was monitored, and the total phenolic content (Folin–Ciocalteu), volatile organic compounds (HS-SPME-GC-MS), together with a sensory evaluation on the overall quality (44 nontrained panelists) were measured for all products at the end of fermentation. A higher total phenolic content resulted in honeydew honey meads, as well as the correspondent honey wine prepared with black currant. A total of 46 volatile organic compounds for pre-fermentation samples and 62 for post-fermentation samples were identified belonging to higher alcohols, organic acids, esters, and terpenes. The sensory analysis showed that the difference in meads made from blossom honey and honeydew honey was perceptible by the panelists with a general greater appreciation for the traditional blossom honey mead. These results demonstrated the influences of different components in meads, in particular, the influence of honey quality. However, further studies are needed to establish the relationship between the chemical profile and mead flavor perception.

## 1. Introduction

Mead, also called honey wine, is traditionally an alcoholic beverage obtained through yeast fermentation of diluted honey. Mead is found in the history of many countries all around the world, and it is one of the oldest alcoholic beverages with variable alcohol content (8–18% alcohol *v*/*v*), mostly depending on the honey to water dilution ratio. Besides the traditional mead (the fermented diluted honey), many variations can be found, containing also herbs and spices (metheglin) or fruit (melomel) [1].

Fructose and glucose are generally the most abundant simple sugar found in honey, and fructose is the dominant one (on average from 32% to 42% depending on the honey origin [2]). The mead fermentation process is usually longer than most alcoholic fermentation, where other sugars are present and in higher concentrations. In fact, this fermentation often takes several months to complete, depending on the type of honey, yeast strain, and honey-must composition. Mead contains ethanol and many other compounds, such as sugars, acids, vitamins, phenolic compounds, and minerals, also in dependence on the added ingredient beside honey (reviewed [3]).

The three main factors mead flavor depends are the honey, the yeast strain carrying out the alcoholic fermentation, and the fermentation conditions. Flavor perception may also be influenced by the final alcohol content, the residual sugars, and the acidic value.

Besides the botanical and the geographical origin, honey can be divided into two main groups: blossom or floral honey and honeydew honey. The former is produced starting from nectar from the flower of blossoming plants, while the latter from the exudates from certain plants (such as *Pinus, Abies, Castanea*, and *Quercus*, among others), usually with the concourse of insects, mainly from the family Aphididae [4]. Honeydew has a stronger taste than blossom honey and is perceived as less sweet. It has higher antioxidant activity and a higher concentration of oligosaccharides. Many researchers have found out that honeydew honey with a darker color has a higher concentration of total phenolic compounds and a higher antioxidative capacity [5,6]. For honey wine production, wine yeast strains are usually used because the sugar, pH, and nitrogen characteristics in mead are similar to the ones of grape must [1]. Yeast produces during fermentation many metabolites, which have a large impact on the beverage flavor. Most of the unique flavors of mead depend on the type of honey. For every additional ingredient, additional flavor molecules may be developed [7]. The formation of metabolites during fermentation not only depends on the raw materials used but also on the yeast strain. Most by-products are synthesized when there is a high rate of sugar and another nutrient uptake by yeasts, which all join the catabolizing pathway at the level of pyruvate. Many chemically different metabolites in diverse concentrations contribute to the flavor of alcoholic beverages, such as organic acids, fusel alcohols, aromatic alcohols, esters, carbonyls, and various sulfur-containing compounds [8,9]. Yeast activity behavior during fermentation caused by stressful conditions can lead to the production of unwanted flavors, for example, high volatile acidity or undesired esters [10].

The acids in the mead are coming from the honey, the added fruits, and the acids used for acidification of the must [11]. The acids in honey are usually citric, malic, succinic, formic, proglutamic, acetic, gluconic, and lactic [12]. Organic acids have a very important function in alcoholic beverages influencing organoleptic characteristics and product stability. The addition of organic acids is regulated by the European legislation for wine and recommended because low pH helps to minimize the risk of microbial spoilage by preventing bacterial growth. On the other hand, pH values below 3 could make a stressful environment for the yeast, which leads to the production of undesirable by-products [13].

Volatile organic compounds (VOCs) in mead are present due to raw materials or produced by yeast during fermentation. VOCs belong to various chemical classes, such as esters, higher alcohols, acids, aldehydes, ketones, etc. They have an impact on the aroma and odor of mead and especially contribute to the fruity and floral nuances [7]. Alcohols, such as n-propanol, iso-butanol, 2-phenylethanol, amyl alcohol, and others, influence the flavor of alcoholic beverages. In high concentrations, their flavor is usually described as a solvent, and they contribute to the intensification of an alcoholic taste, which creates a warm mouth feeling [8,14]. In lower concentrations, they may have a positive effect, increasing the complexity of fermented beverages [15]. Esters contribute to a floral and fruity flavor of mead. Ethyl acetate, for example, is considered as fruity or solvent (depending on concentration and combination with other volatiles), and isoamyl acetate has a banana or apple flavor. Ethyl esters are the most present in mead because ethanol is the most available substrate [8]. Terpenic compounds are mostly produced by plants and some insects but also from yeast [16] and have been found in remarkable concentrations in mead [7,13,17]. Organic acids have a sour flavor, and additionally, they can have individual characteristic flavors. Short-chain fatty acids have a mostly negative influence on the flavor, depending on the concentration and combination of the product. Furthermore, they affect foam performance [8]. In the mead fermentation process, acetic acid and succinate acid are formed in considerable amounts. They reduce the pH, increase the total acidity, and reduce the dissociation of fatty acids. High amounts of acetic acid, succinic acid, and a high concentration of fatty acids may cause a slowdown of the fermentation process [18].

Despite the long tradition of mead making, a limited scientific background is available in this field, which may be due to the medium and small-scale production. Therefore, systematic information and knowledge are needed in order to be able to define those parameters necessary to understand how to master mead quality, develop adequate formulations, and optimize the fermentation conditions as reviewed by Iglesias et al. [3].

The present work aimed to contribute to filling some of the gaps on the influence of different ingredients used for mead making on the final flavor profile. To reach this aim, multi-floral blossom honey and a forest honeydew honey, with or without black currant (*Ribes nigrum*), added before fermentation, were used to prepare four different honey wines to be compared for their volatile organic compounds content. Due to the high complexity of the *Ribes nigrum*, its addition was decided to originate a mead variant rich in polyphenols, minerals, and vitamins, which might have a positive effect on the fermentation process or the end product quality [19]. Polyphenol content and consumer acceptance were explored, as well.

## 2. Results and Discussion

Four different meads of honey wine were prepared either with multi-floral blossom honey and forest honeydew honey alone (B and H, respectively) or with black currant added (BC and HC, respectively).

### 2.1. Fermentation Kinetics

Fermentation kinetics were monitored by measuring the weight loss due to CO_2_ effluence. HC was the first product reaching the stationary phase in fermentation after approx. 14 days, whereas H and BC took approx. 20 days. B had the longest fermentation time with approx. 30 days (Figure S1). According to these results, the products prepared with blossom honey had a longer fermentation time, and the addition of black currants accelerated the fermentation. Differences in honey composition [20] and black currant [19] might provide factors influencing the yeast rate of sugar depletion.

Total CO_2_ production in g/L was equal to 86.1 (±0.3), 90.6 (±0.7), 75.5 (±0.2), and 79.2 (±0.5) for B, BC, H, and HC, respectively, in theory corresponding to 176.3 (±0.7), 185.5 (±1.4), 154.5 (±0.5), and 162.1 (±0.9) of fermented glucose in g/L. Measurement of sugar content in the original honey showed 690 ±10 g/L for the blossom honey and 650 ±10 g/L for the honeydew honey, expressed as total glucose (sum of glucose, fructose, and sucrose contribution). Glucose and fructose measured in the prepared product before fermentation showed a lower amount of glucose and fructose in H and HC (177.0 and 177.1 g/L, respectively), compared to B and BC (202.5 and 201.5 g/L, respectively). Further measurement of honey sugars besides the one measured might provide a more detailed explanation.

Being the residual sugar content in all honey wines below 5 g/L (Table 1), even if differences in fermentation length were observed, all the fermentation could be considered successfully completed.

### 2.2. Physiochemical Parameters

The mean values for all parameters measured in the fermented product are listed in Table 1. B, which had a residual sugar value of 4.3 ± 0.6 g/L, significantly differed from the others, although usually meads can be considered dry [11]. Ethanol concentration in the four products was 11.32% *v*/*v*, 10.63% *v*/*v*, 8.60% *v*/*v*, and 8.66% *v*/*v*, respectively, measured in B, BC, H, and HC. Ethanol content was significantly higher in varieties with blossom honey. Even if prepared at a similar honey dilution rate, blossom honey used in B and BC products had a higher fermentable sugar content, explaining both the higher ethanol content and the higher carbon dioxide loss observed in the B and BC products.

The pH did not change to a great extent during the fermentation, and the lower one was recorded for the B mead, and L-lactic acid content was not significantly different in the four products. Acetic acid content at the end of fermentation ranged between 0.20 g/L and 0.40 ±0.02 g/L. Besides being yeast strain-dependent and related to the amount of sugar fermented, acetic acid might be also providing information on the yeast stress status or be the symptoms of microbial spoilage. This latter event seemed not to have occurred in the investigated products, and the acetic acid was detectable in amounts comparable to previous studies [3,13,18]. Acetaldehyde concentration ranged between 6.2 ± 1.2 and 16.8 ± 2.2 mg/L. Acetaldehyde concentration in meads usually ranges between 18.2 and 125.5 mg/L, as reported in the literature [21]. In general, 0.5–286 mg/L is the concentration of acetaldehyde produced by *Saccharomyces cerevisiae* in white wine [22]. A maximum of 0.5 g/L has been indicated in beer [8]. High concentrations of acetaldehyde would lead to a pungent, green, and grassy flavor, and it is associated with microbial spoilage of the fermented beverages. The acetaldehyde concentration found in this study testified the absence of microbial spoilage. Lactic acid was measured in very few quantities of 0.14–0.36 g/L. This was an indication that no lactic fermentation from bacteria took place.

### 2.3. Total Polyphenolic Content in Honey Wine

Total polyphenolic content measured using Folin–Ciocalteu reagent was 54.91 mg/L (±2.16) in B honey wine, 289.09 mg/L (±14.97) in BC, 101.95 mg/L (±5.78) in H, and 304.44 mg/L (±14.13) in HC, all expressed as mg/L of gallic acid. Results for traditional meads (B and H) were similar to the results reported in the literature [23,24]. Blossom honey varieties had less polyphenolic content than honeydew honey varieties, which corresponded to the result found in the literature [5,6]. Products prepared with currants (BC and HC) had much higher concentrations than the traditional meads as expected. The black currants added about 200 mg/L of polyphenols to the mead. Together with the red color, black currant imparted to the honey wine a higher capacity to counteract oxidation, due to the higher amount of compound recognized to protect from oxidation [25] and as found in polish mead by Socha [26].

### 2.4. Volatile Organic Compounds

Meads, fermented from honeydew honey and blossom honey with and without the addition of *Ribes nigrum,* were analyzed to evaluate the influence of the starting honey and blackcurrant addition on the volatile organic compounds profile using HS-SPME-GC-MS method. The flavor and aroma of the final product depend on the type of honey and the floral source, the fermenting yeasts and conditions, and the presence of additives and fruits [27,28,29].

In this work, a semi quantification of 62 compounds was reported as an average of three biological replicates (Table S1); the table includes the compound name and class, Chemical Abstracts Service (CAS) number, retention index, retention time, and level of identification. Peaks in the chromatograms, acquired in full scan mode, had been integrated and reported as the area ratio of the peak with the internal standard (2-octanol). An ANOVA test was performed with a Turkey posthoc method to identify significant differences between the samples. In total, 4 acids, 13 alcohols, 4 aldehydes, 14 esters, 1 ketone, 16 terpenes and derivatives, 7 others, and 3 unknown compounds were identified. A general view of the results showed a higher compound formation or increase in samples analyzed after the fermentation.

**Figure 1:**
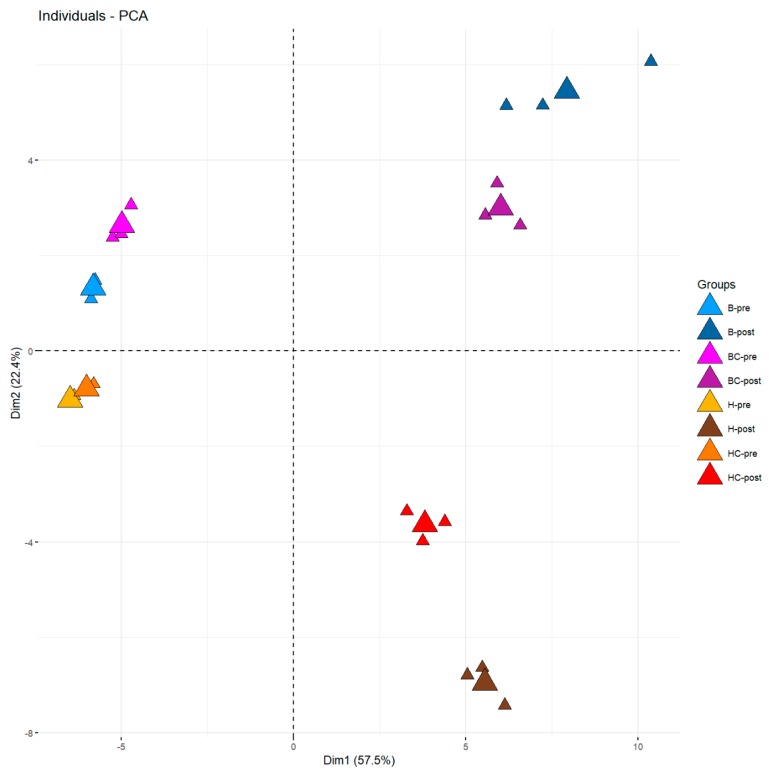
Principal component analysis (PCA) of the volatile compounds found in four honey wines made with blossom honey (B), blossom honey with blackcurrant (BC), honeydew honey (H), and honeydew honey with blackcurrant (HC), analyzed before (pre) and at the end of the fermentation process (post). The first component explained 57.5%, and the second component explained 22.4% of the total variance. Samples replicates are shown by the smaller triangle-shaped dots, while the average is represented by the bigger shaped dots.

**Figure 2:**
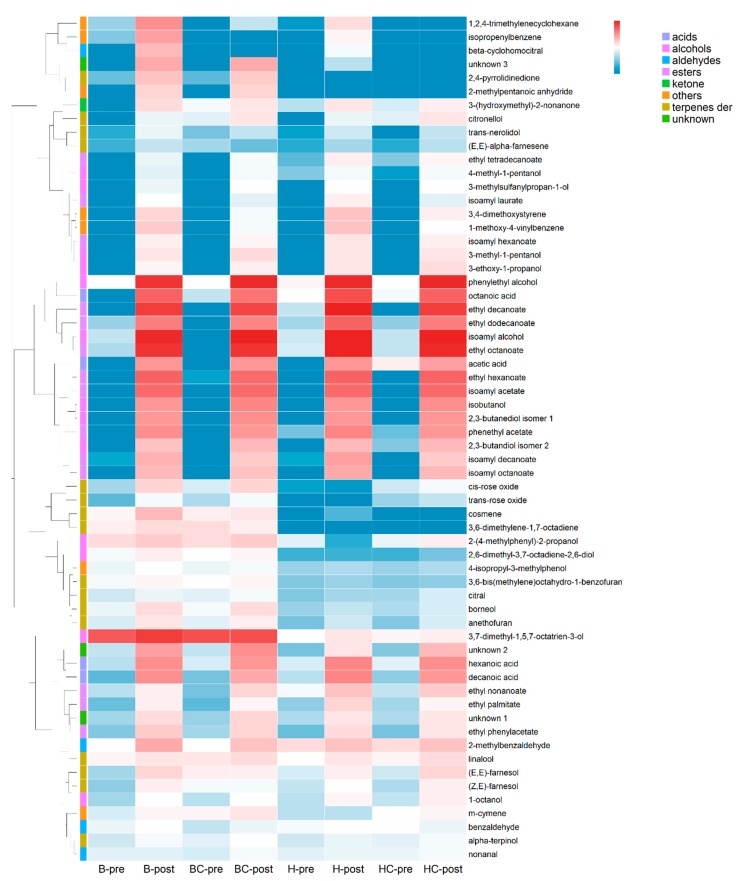
Heatmap and one-dimensional hierarchical dendrogram of the volatile compounds found in the investigated samples. Heatmap represents a log10 transformed data (average of three replicates). The heatmap color represents the magnitude of each compound. Dark red color indicates the higher magnitude, and then the magnitude gradually decreases to light red, white, light blue, up to dark blue (one order each), with the latter, indicating the lower magnitude. The magnitude represents transformed data (log10 of the ratio) to fit them in the same range. On the left: the colored sidebar indicates the class of metabolites. Sample legend: B = blossom honey, BC = blossom honey with blackcurrant, H = honeydew honey, HC = honeydew honey with blackcurrant, pre = pre-fermentation, and post = post-fermentation.

To explore the dataset, a principal component analysis (PCA) was performed using the three replicates for each mead (Figure 1). The first and second components explained 80% of the total variance; the eigenvalues and the correlation results between variables and PCs are reported in Supplementary File 1. The first component allowed the separation between pre-fermentation and post-fermentation samples; the second component allowed to separate meads based on the honey (honeydew honey or blossom honey) and the blackcurrant addition. To better understand which metabolite influenced the diversification of the products, a heatmap was used (Figure 2) with a log10 transformed data (average of three replicates).

#### 2.4.1. Volatile Profile in the Products before and after Fermentation with *S. cerevisiae*

Looking at the PCA, a clear separation between the pre- and post-fermentation samples was noticeable due to the volatile organic compounds produced during the fermentation. The number of identified VOCs increased in post-fermentation samples since some VOCs are produced by the yeasts during the alcoholic fermentation [30]. At least 18 compounds produced by the yeasts during the fermentation process were found, being mainly alcohols and esters (Figure 2), already described as the products of *S. cerevisiae* EC1118 fermentation [31,32]. As reported in the literature, it is well known that yeasts are VOCs producers; in wine, the main groups of compounds that form the fermentation bouquet are the acids, alcohols, and esters and, to a lesser extent, aldehydes and ketones [33].

**Figure 3:**
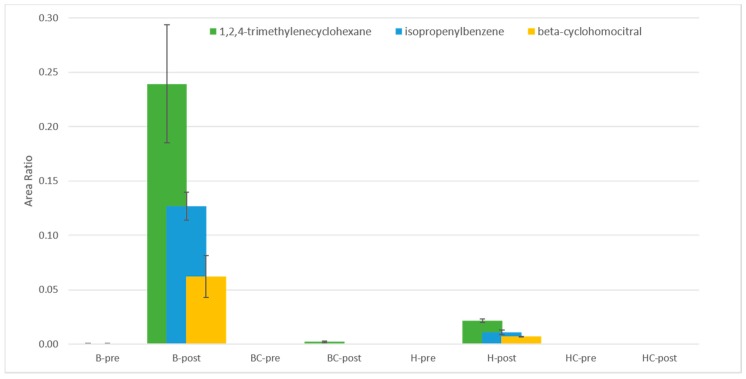
1,2,4-trimethylencyclohexane, isopropenylbenzene, and beta-ciclohomocitral in honey wine before and after fermentation by *S. cerevisiae* EC1118. Samples were named as follows: B = blossom honey, BC = blossom honey with blackcurrant, H = honeydew honey, HC = honeydew honey with blackcurrant, pre = pre-fermentation, and post = post-fermentation.

Furthermore, it is known that the compounds influencing the aroma of alcoholic beverages are mainly higher alcohols, esters, volatile acids, and aldehydes [34], making yeasts strain the main actor in establishing the sensory characteristics. Looking at our results, B and BC post-fermentation had a higher number of volatile compounds than H and HC post-fermentation. Being that the yeast strains are the same in all fermentation processes, the different numbers are due to the different starter matrix. In the product B-post, 62 VOCs were detected, while BC-post was characterized by 60 different compounds. In H-post and in HC-post, 56 and 54 VOCs were, respectively, identified. The product prepared with blackcurrants, in pre- and post-fermentation, had a lower VOCs content than those produced with the same honey but without fruit addition. The compounds that contributed to this difference were 1,2,4-trimethylenecyclohexane found in B-post and H-post and a very low amount in BC-post, isopropenylbenzene found in B-post and H-post, and beta-cyclohomocitral only found in B-post (Figure 3). These three compounds were not confirmed by the standard injection; for this reason, their identification could be only considered as putative. It is reasonable to assume that *Ribes nigrum* added the nutrients, shaping the yeast metabolism; however, there is no evidence in the literature that which metabolic pathway leads to the synthesis of these compounds that might be regulated during fermentation by yeast.

**Figure 4:**
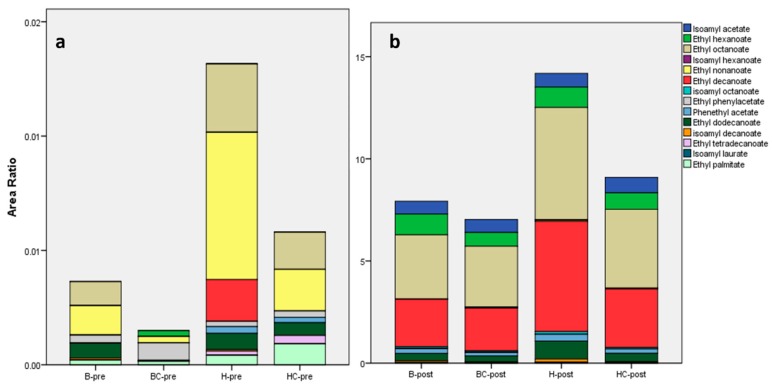
Esters composition in pre (**a**) and post (**b**) fermentation samples. Values represent the area ratio with the internal standard (n = 3). B-pre: blossom honey pre-fermentation; B-post: blossom honey post-fermentation; BC-pre: blossom honey with blackcurrant pre-fermentation; BC-post: blossom honey with blackcurrant post-fermentation; H-pre: honeydew honey pre-fermentation; H-post: honeydew honey post-fermentation; HC-pre: honeydew honey with blackcurrant pre-fermentation; HC-post: honeydew honey with blackcurrant post-fermentation.

The post-fermentation volatile organic compounds had been highlighted in the heatmap (Figure 2). Among them, isoamyl alcohol was found featuring in many fermented alcoholic beverages and already reported in mead [10,17,35], giving a solvent, sweet, and nail polish aroma and also part of fusel oil [36]. Another detected fusel oil representative was isobutanol, only found in post-fermentation samples (Figure 2), which lead to green notes in the flavor of beverages [37]. Esters are contributing to the fruity and floral nuances of meads [38]. In the samples, 14 esters were found; these compounds appearing in different concentrations in the samples, as shown in Figure 4, with a visible magnitude increased at the end of the fermentation process. H-pre had a higher content of esters and a slightly different profile of this class of compounds compared with the other pre-fermentation samples (Figure 4a). H-pre was, in fact, characterized by the presence of ethyl octanoate end ethyl nonanoate in higher amount and by the presence of ethyl decanoate not revealed in the other worts. In the same product, at the end of the fermentation process, the esters profile seemed to be more similar, although H-post exhibited a higher content in ethyl octanoate and ethyl decanoate compared to the other honey wines (Figure 4b). All found esters are common components in alcoholic beverages and are often found in fruits. In detail, the most abundant compounds determined in our fermented samples were isoamyl acetate, a characteristic compound of banana flavor, ethyl hexanoate and ethyl octanoate that contribute to a sweet and strawberry-like aroma, and ethyl decanoate with a sweet, fruity apple flavor [39]. Ethyl octanoate and ethyl decanoate aroma have also been described as waxy and soapy [40].

#### 2.4.2. Honey Influence on the Aromatic Characteristics of the Product

The honey used for the fermented product represented another important factor influencing the final aroma of the beverages. In our study, two types of honey were used: blossom honey (B) and honeydew honey (H). The PCA showed a separation on the second component, based on the honey used for the fermented beverage (Figure 1); looking at the heatmap, we identified some compounds potentially related to the types of used honey (Figure 2).

Few compounds seemed to be higher in B-pre product compared to the others and were accumulated in B-post products in comparison to the H samples; they were 2-(4-methylphenyl)-2-propanol, 2,6-dimethyl-3,7-octadiene-2,6-diol, 3,6-bis(methylene)octahydro-1-benzofuran, 3,6-dimethylene-1,7-octadiene, 3,7-dimethyl-1,5,7-octatrien-3-ol, 4-isopropyl-3-methylphenol, citral, and m-cymene.

This cluster showed to be more abundant in all B meads (with and without blackcurrant addition) compared with H samples. Other characteristic compounds of B meads were *trans*-nerolidol, *cis* and *trans*-rose oxide, anethofuran, borneol, and ethyl phenylacetate. These compounds produced during the fermentation process (Figure 2) could be responsible for a floral, rose, and balsamic camphor perception in the final product. Ethyl phenylacetate can not only contribute to a positive note, but it is also considered as off-flavor formed in beer during aging from precursors, which are produced during the fermentation [41].

Regarding the honeydew honey samples, no typical compounds were found in pre-fermentation samples. However, the final beverage seemed to be richer in acids, such as hexanoic acid, octanoic acid, decanoic acid, and esters, such as ethyl octanoate, isoamyl hexanoate, ethyl nonanoate, ethyl decanoate, ethyl dodecanoate. Short-chain fatty acids, such as octanoic acid (caprylic acid) and hexanoic acid (caproic acid), were also reported [17]. These compounds were present in higher concentrations and are associated with negative characteristics of “rancid,” “cheese,” and “fatty” aroma [7].

Terpene compounds were found in both pre- and post-fermentation samples, and, as expected, these compounds mostly originate from the raw materials. Many of the detected terpenes are known for their positive influences on mead aroma. Citral, for example, has a lemon-like pleasant odor [42], linalool a floral and spicy odor [43]. The stereoisomers *cis*-rose oxide and *trans*-rose oxide are found in flowers, and fruit and essential oils can contribute to fruit and floral notes in fruits and grapes. Mentofuran is a constituent of peppermint oil [44].

#### 2.4.3. Influence of *Ribes nigrum* Addition

Blackcurrant addition, with its peculiar composition, had the capability to modify the fermentation environment for the yeast, leading to different volatiles, influencing, therefore, the aroma profile of the final product. Blackcurrant aroma is characterized by various volatile components, including esters and terpenoids. As reported, cultivars and growing and storage conditions can affect the flavor component [45,46]. Among the most reported compounds for the characteristic of blackcurrant fruit, 2-methylbutyl acetate, methyl butanoate, ethyl butanoate, and ethyl hexanoate, belonging to the esters class, that confer fruity and sweet notes are mentioned. Besides, nonanal, beta-damascenone, and monoterpenes, ketones, and sulfur compounds, such as 4 methoxy-2-methyl-butanethiol (catty note flavor), are reported. In our results, we found few compounds with significantly higher amounts in BC and HC post-fermentation: citronellol, a-terpinol, and nonanal, as shown in Figure 2. It was noted that besides the esters nonanal, an aldehyde C-9 could give the classical aldehyde note of waxy, citrus, floral, and green.

### 2.5. Sensory Test

The sensory test was carried out in order to find a possible link between the volatile compound profile of the product and their sensory properties (Table S2). Among the 44 nontrained participants to the sensory panel, 77% correctly paired the honey wine prepared with the same honey; the sensory differences linked to the honey used for mead production were, therefore, significantly perceivable.

A nine-point hedonic evaluation scale was structured, point 1 stated for “dislike extremely”, while point 9 stated for “like extremely”. Using this scale, panelists described the samples as likable as on average; most judged the product as like slightly, like moderately, or like very much. There was no significant difference between the samples in relation to the overall impression and no significant difference between the samples in relation to the olfactory impression. The acceptance level for the honey wine for the sensorial analysis was expressed as a mean value. None of the means presented significant differences. Overall impression and flavor attributes for all beverages variated from 64 to 68% and between 65 and 72%, respectively. According to Dutcosky [47], the acceptance factor (AF) ≥ 70% represented good acceptability for the attribute analyzed in a sensorial analysis. Honey wine obtained with blossom honey showed an AF of 72%.

Honey wine B ranked first, followed by BC, HC, and H, for both the overall impression and the odor. According to the Friedman test, only the ranking for the odor impression showed to be significant. This seemed to be in line with the AF. This might be related to the higher amount of hexanoic acid, octanoic acid, decanoic acid, or esters, such as ethyl octanoate, isoamyl hexanoate, ethyl nonanoate, ethyl decanoate, ethyl dodecanoate found in H and HC product compared to B and BC. Short-chain fatty acids, such as octanoic acid (caprylic acid) and hexanoic acid (caproic acid), have been associated with negative characteristics, such as “rancid,” “cheese,” and “fatty” aroma [7]. The odor of esters like ethyl octanoate and ethyl decanoate odors have also been described as waxy and soapy [40].

In general, it seems that the VOCs profile imparted by the honey, described above, also has some impact on the sensory perception. However, for a more descriptive sensory evaluation, a trained panel will be able to provide a clearer link between the sensory perception and the specific volatiles or group of volatile as distinguished in the heatmap.

## 3. Materials and Methods

### 3.1. Mead Ingredient

Honeydew honey (Bosco, Mieli Thun, Vigo di Ton, Italy) and blossom honey (mixed honey with a prevalence of *Ailanthus altissima*) (Mieli Thun, Vigo di Ton, Italy) were used. Honeydew honey was collected in the northern Italian wood; dark amber color was described by the supplier as spicy (black pepper, juniper berries, and cloves) with a note of fresh vegetables, carob, rhubarb, and liquorice stick. Blossom honey had a golden color and a creamy consistency and was characterized, according to the supplier, by the smell of muscat grapes and peach syrup; lychee was conferred by the prevalence of *Ailanthus altissima*, also known as the tree of heaven. One part of honey (*w*/*w*) was used in all the preparation. Warm tap water was used to dissolve the honey. Black currant (*Ribes nigrum*), common berry fruit in South Tyrol, Italy, had been purchased from a local producer and stored at −80 °C until used. Half part of berry fruit (w/w) was used for the *Ribes nigrum* added recipes. The berries were crushed before the addition of other ingredients. The final mixture had a temperature of about 30 °C before acidification. Acidification was carried out using a citrate buffer in order to reach a pH value between 3–3.2. A preliminary test was carried out to establish the amount of citrate buffer to be added to each mixture.

*Saccharomyces cerevisiae* yeast strain EC1118 (Lallemand Inc., Montreal, QC, Canada) was used in the dry active form at the ratio of 25 g/hl after rehydration, according to the manufacturer’s instructions. As a yeast protectant in the rehydration step (GoFerm Lallemand Inc., Montreal, QC, Canada), it was used at a 1:1 ratio with the weighted yeast. This product contained all essential vitamins, minerals, and amino acids required to create a non-stressful environment for yeast rehydrating in water.

To ensure the necessary amount of nitrogen and avoid a stuck fermentation, “FermaidE” (Lallemand Inc., Montreal, QC, Canada) was added to the must as a vitamin, organic, and inorganic nitrogen source at the ratio of 30 g/hl.

### 3.2. Honey Wine Wort Preparation and Fermentation Follow Up

Four different recipes for honey wine preparation were used. Blossom honey with and without black currant recipes was compared with honeydew honey with and without black currant (B, BC, H, and HC, respectively). Each recipe was tested in triplicate, carrying out the fermentation in a 5 L glass flask filled up to 3.5 L. All flasks were closed with an air-lock valve. All recipes are described in Table 2. Nitrogen exogen source was added, according to the manufacturer’s instructions: two-third at the yeast inoculum and the remaining one-third after one-third of the fermentation was completed. Fermentation was monitored, measuring the weight loss once or twice per day. All the flasks were incubated at 18 °C. Fermentation end was detected when two subsequent weight measurements did not differ for more than approx. one gram: at this stage, flasks were left overnight at 4 °C. After overnight cold storage, berry solids were separated with the aid of a strainer, honey wine was separated by the yeast sediment, and samples were collected for further analysis. The remain was bottled and left at 4 °C until the sensory test. Samples were immediately analyzed for pH and total acidity. For the other analyses, samples were stored at −80 °C until used.

### 3.3. Honey Wine Analysis

#### 3.3.1. Physicochemical Parameters

The total soluble solids determined as Brix were measured using a digital refractometer (PAL-BX/RI, Atago, WA, USA). For the pH, a pH electrode was used (pH70+ DNH pH meter with Digital pH electrode mod 201 T, XS instruments, Carpi, Italy).

The content was the sum of glucose and fructose after inversion was measured, according to the OIV-MA-AS311-02 R2009 + OIV-MA-AS2-03B R2012. The alcohol content in volume percent (% vol) was measured following the international methods of wine and must analysis (OIV-MA-AS312-01A R2016 par 4.B). Fructose, glucose, and sucrose in honey were expressed as g/L of glucose, acetic, and lactic acid, and residual sugars were measured enzymatically with the CDR BeerLab^®^ Touch (CDR s.r.l., Ginestra Fiorentina, Italy), according to the manufacturer’s instructions.

Acetaldehyde content was measured with the aid of a spectrophotometric-enzymatic-based kit for acetaldehyde, according to the manufacturer’s instructions (K-ACHYD 06/18, Megaenzyme International Ireland INC. Bray, Co. Wicklow, Ireland), measuring the absorbance variation at 340 nm with the aid of a microtiter plate reader spectrophotometer (Multiscan Sky Spectrophotometer, Thermo Fisher Scientific Life Technologies Italia, Monza, Italy) where a 96-well microplate was used.

#### 3.3.2. Total Polyphenolic Content

The polyphenolic content of the meads was measured using Folin–Ciocalteu reagent (Merck KGaA, Darmstadt, Germany) in a colorimetric assay. The method was adapted from a published method [48]: Folin-Ciocalteu reagent was added to the sample, and after a reaction time of 3 min at room temperature, 2 M sodium carbonate solution was added. After two hours at 21 °C, the absorbance at 765 nm was read. For the quantification, a standard curve ranging from 50–500 mg/L of gallic acid was prepared. The polyphenol content was determined by linear regression from the standard curve, and the results were expressed as mg/L of gallic acid.

### 3.4. Volatile Organic Compounds

For the volatile organic compounds (VOCs) analysis, a headspace solid-phase microextraction coupled with gas chromatography-mass spectrometry (HS-SPME-GC-MS) was used (QP2010 SE Shimadzu, Kyoto, Japan). The fermented mead, as well as the unfermented musts, were examined. The method was adapted from Ravasio et al. [49]. Briefly, 2.5 mL of sample was placed in 20 mL glass vials with 0.75 g of NaCl and 10 µL of internal standard (2-octanol, 50 µg/mL). Samples were incubated for 10 min at 40 °C and 250 rpm. Compounds in the headspace were adsorbed for 40 min at 40 °C using 2 cm DVB/CAR/PDMS 50/30 µm fiber from Supelco (Bellefonte, PA, USA). Compounds were desorbed in the GC inlet at 250 °C for 4 min. Chromatographic separation was carried out using a ZB-WAX (30 m × 0.25 mm × 0.25 µm, 40–260 °C) Capillary GC-Column Zebron (Phenomenex, Torrance, CA, USA), using helium as carrier gas at 1.2 mL/min.

The temperature program for the oven was set as follows: 40 °C for 4 min, then up to 250 °C, at 6 °C/min held for 5 min. The total run time was 44 min. The mass spectrometer (quadrupole) was operating in full scan mode, detecting fragments in a mass range of 35 to 350 m/z. Data processing was performed using GC-MS solution Software from Shimadzu. The most intensive ion was used as a quantifier and the ratio of the second and third as a qualifier. The identification of VOCs was carried out by comparing mass spectra using the NIST 2017 database, retention indexes, and with the standard injection when available. The experimental linear temperature retention index of each compound was calculated using a series of n-alkanes (C8-C20) in the same experimental conditions as the samples. Results were expressed as area ratio between compound and internal standard.

### 3.5. Sensory Analysis

A sensory consumer test was carried out with the aid of 44 nontrained consumer panelists; each participant was asked to evaluate all four types of honey wine. Forty milliliters of samples of each product were served for each panelist. Samples were coded with the number served in different sequences and arrangements randomly. The panelists were asked to rate each sample, with a nine-point hedonistic scale, for the (a) the overall taste impression and (b) for the odor overall impression. The nine-point scale was structured as follows: 9: like extremely, 8: like very much, 7: like moderately, 6: like slightly, 5: like neither nor, 4: dislike slightly, 3: dislike moderately, 2: dislike very much, 1: dislike extremely. The acceptability of the tested beverages was assessed by calculating the acceptability factor (AF) using standardized criteria: AF = A*100/B, where A is the mean value obtained for each attribute, and B is the maximum value used to judge each attribute [47,50]. They were asked also to pair the product made with the same honey. Participants were asked to rank the four samples for both the overall impression and the odor overall, assigning the number one to the best one and number four the worst.

### 3.6. Statistical Methods

For statistical analyses, SPSS Statistics software Version 26 (IBM Inc., Armonk, NY, USA) and Microsoft Excel 2019 software were used. Means for every data are expressed as arithmetic mean ± standard deviation of the three replicates for every product. To determine if there was a significant difference between results, a one-way analysis of variance (ANOVA) and a Tukey posthoc test were performed. For all analyses, *p* < 0.05 was considered statistically significant. Used test and corresponding *p*-value were indicated together with the result in each specific session. In addition, Microsoft Excel 2019 was used to verify the significance of the pairing test [51]. Friedmann-Test for statistical analysis with n = 44 test subjects and k = 4 samples was carried out using Microsoft Excel 2019 [48]. The R FactoMineR package was used to perform the PCA [52], and the factoextra package for extracting and visualizing the results. The data had been scaled to unit variance before the analysis to avoid some variables to become dominant just because of their large measurement units. The NMF package was used for the heatmap and hierarchical cluster analysis with a Euclidean distance [53].

## 4. Conclusions

Two different kinds of honey in the presence or absence of black currant were tested for honey wine production. Using these ingredients, no stressful condition seemed to be occurring for the yeast, leading to fermentation delay or arrest, producing a medium or low alcoholic drink. The fermented products were described by a large number of volatile organic compounds capable of allowing the distinctive metabolic contribution of the yeast, also as a response to the honey and the fruit added in fermentation. In particular, the honey contributed to shaping a specific volatile profile, somehow perceivable during sensory analysis. To a lesser extent, also using berry fruit, such as black currant, provided a way to shape flavor and polyphenols content of the final drink. Further investigation would be necessary to evaluate the specific sensory contribution of every single volatile organic compound alone or in association with others found in this study. More information on volatile metabolites associated with mead had been provided that might help to develop alternative medium to low alcoholic drinks at a reasonable cost, adding value to beehive products.

## Figures and Tables

**Table 1:** Physiochemical parameters characterizing the four products prepared with blossom honey (B), blossom honey and blackcurrant (BC), honeydew honey (H), and honeydew honey and black currant (HC). Parameters were measured before fermentation (t0) and/or at the end of the fermentation process (END). END data are reported as the average of the three biological replicates ± standard deviation (in brackets). Apex letter in the same row shows the results of the statistic evaluation, and different letters correspond to significant different parameters (*p* ≤ 0.05).

	Analysis Time	B	BC	H	HC
pH	t0	3.17	3.15	3.16	3.22
pH	END	3.13 ^(±0.03) a^	3.33 ^(±0.01) b^	3.27 ^(±0.01) bc^	3.29 ^(±0.01) c^
Brix (%)	t0	21.6	22.9	21.7	21.5
Glu+Fru (g/L)	t0	202.5	201.5	177.0	177.1
Residual sugar (g/L)	END	4.3 ^(±0.6) a^	0.4 ^(±0.1) bc^	1.0 ^(±0.1) b^	0.1 ^(±0.1) c^
Ethanol (% vol/vol)	END	11.32 ^(±0.44) a^	10.63 ^(±0.67) a^	8.60 ^(±0.13) b^	8.66 ^(±0.18) b^
Acetic acid (g/L)	END	0.40 ^(±0.02) a^	0.26 ^(±0.02) a^	0.32 ^(±0.01) a^	0..20 ^(±0.01) a^
Acetaldehyde (mg/L)	END	6.2 ^(±1.4) a^	11.3 ^(±2.1) ab^	10.4 ^(±3.4) ab^	16.8 ^(±2.7) b^
L-lactic acid (g/L)	END	0.14 ^(±0) a^	0.23 ^(±0.02) a^	0.30 ^(±0) a^	0.36 ^(±0.01) a^

**Table 2:** Ingredients used for the four recipes tested for honey wine production. Weight is referring to the total amount of 11 kg prepared for each recipe before aliquoting the 3.5 L of each replicate (n = 3). B: blossom honey; BC: blossom honey with black currant; H: honeydew honey; HC: honeydew honey with black currant.

Recipe	Component	Weight (kg)	Ratio
B	Honey	2.750	1.00
	Water	8.075	2.94
	citrate buffer	0.175	0.06
BC	Honey	2.750	1.00
	Water	6.871	2.50
	Buffer	0	0
	black currants	1.379	0.50
H	Honey	2.750	1.00
	Water	7.750	2.82
	citrate buffer	0.500	0.18
HC	Honey	2.763	1.00
	Water	6.682	2.43
	Buffer	0.180	0.07
	Black currants	1.375	0.50

## Supplementary Materials

The following are available online. Figure S1. Fermentation kinetic; Figure S2. Volatile organic compound measured in the honey wines before and after fermentation; Table S1. Volatile organic compound measurement and descriptions; File S1. Eigenvalues and correlation of the PC analysis; Table S2. Sensory evaluation of the honey wine.
