# Supplementary material for: Volatile Profile of Mead Fermenting Blossom Honey and Honeydew Honey with or without Ribes nigrum
Source: Molecules. 2020 Apr 15;25(8):1818. doi: 10.3390/molecules25081818 (PMC7221654; doi:10.3390/molecules25081818)
Supplement: Supplementary file 1 [file molecules-25-01818-s001.zip › molecules-771489 Figure S1S2 caption.docx]

SUPPLEMENTARY MATERIAL

Figure S1

**
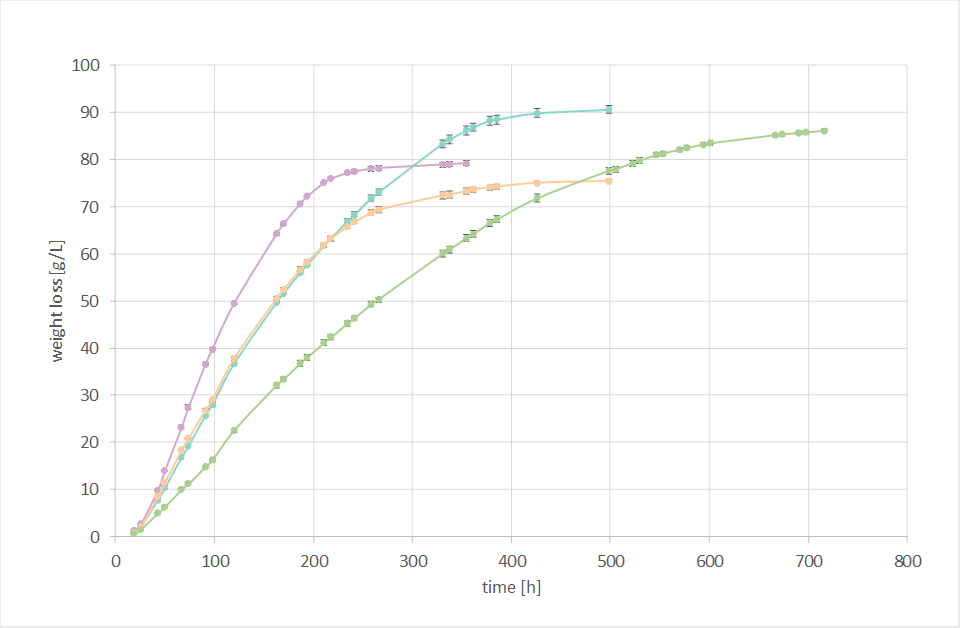
**

Figure S1: Fermentation kinetic revealed during honey wine production in four product prepared with blossom honey (green line and dots), blossom honey with black currant (mint green line and dots), honey dew honey (orange line and dots), and honey dew honey with black currant (mauve line and dots). Fermentation was monitored measuring the weight loss due to the CO2 effluence. Results are shown as average of three replicates, error bars show the standard deviation.

Figure S2

**
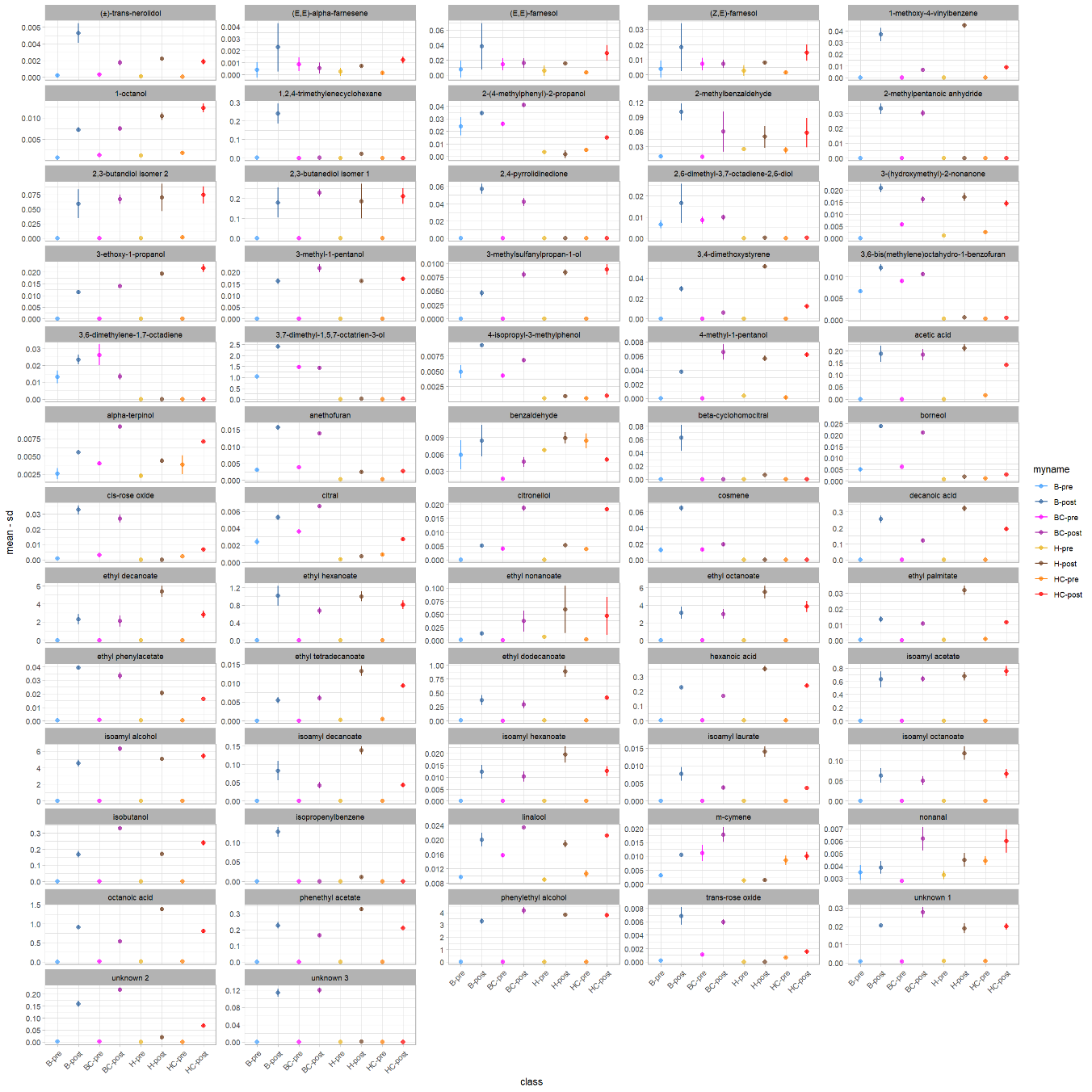
**

Figure S2. Variation of all volatile organic compounds measured during honey wine production in four products prepared with blossom honey (B), blossom honey with black currant (BC), honey dew honey (H), and honey dew honey with black currant (HC). These compounds were analyzed in both pre-fermentation stage (pre) or at the end of the fermentation process (post). Results are shown as average of three replicates, error bars show the standard deviation.
