# Supplementary material for: Volatile Profile of Mead Fermenting Blossom Honey and Honeydew Honey with or without Ribes nigrum
Source: Molecules. 2020 Apr 15;25(8):1818. doi: 10.3390/molecules25081818 (PMC7221654; doi:10.3390/molecules25081818)
Supplement: Supplementary file 1 [file molecules-25-01818-s001.zip › molecules-771489 Table S2.docx]

Table S2 : Sensory description of the four honey wines based on hedonistic scale (HS – average of the score based on the 9 points hedonic scale), acceptance factor (AF - %) and ranking (RK, sum of ranks) for overall impression and odor impression. B: blossom honey; BC: blossom honey and black currant; H: honeydew honey; HC: honeydew honey and black currant.

|  | **HS** | **AF (%)** | **RK** | **HS** | **AF (%)** | **RK** |
| --- | --- | --- | --- | --- | --- | --- |
|  | **overall** | **overall** | **overall** | **odor** | **odor** | **odor** |
| **B** | 6.11 | 68 | 97 | 6.52 | 72 | 97 |
| **BC** | 5.82 | 65 | 111 | 6.05 | 67 | 98 |
| **H** | 5.75 | 64 | 116 | 5.86 | 65 | 124 |
| **HC** | 6.02 | 67 | 116 | 6.07 | 67 | 121 |

For details on the different methods please look at the specific paragraph of section 4 in the main manuscript.
