# Supplementary figures and images for: Volatile Profile of Mead Fermenting Blossom Honey and Honeydew Honey with or without Ribes nigrum
Source: Molecules. 2020 Apr 15;25(8):1818. doi: 10.3390/molecules25081818 (PMC7221654; doi:10.3390/molecules25081818)

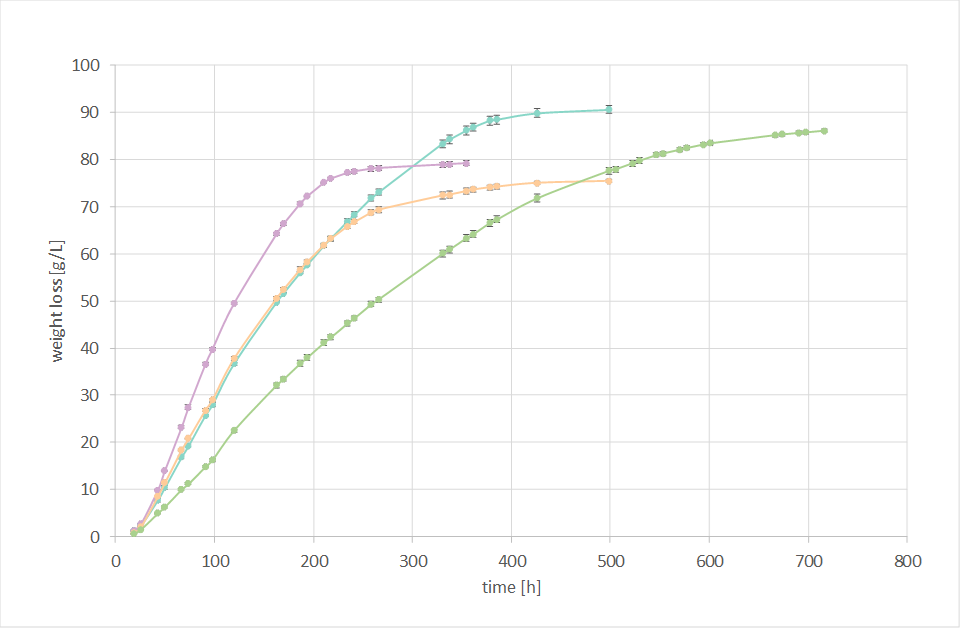

Supplement: Supplementary file 1 [file molecules-25-01818-s001.zip › molecules-771489 Figure S1.tif]

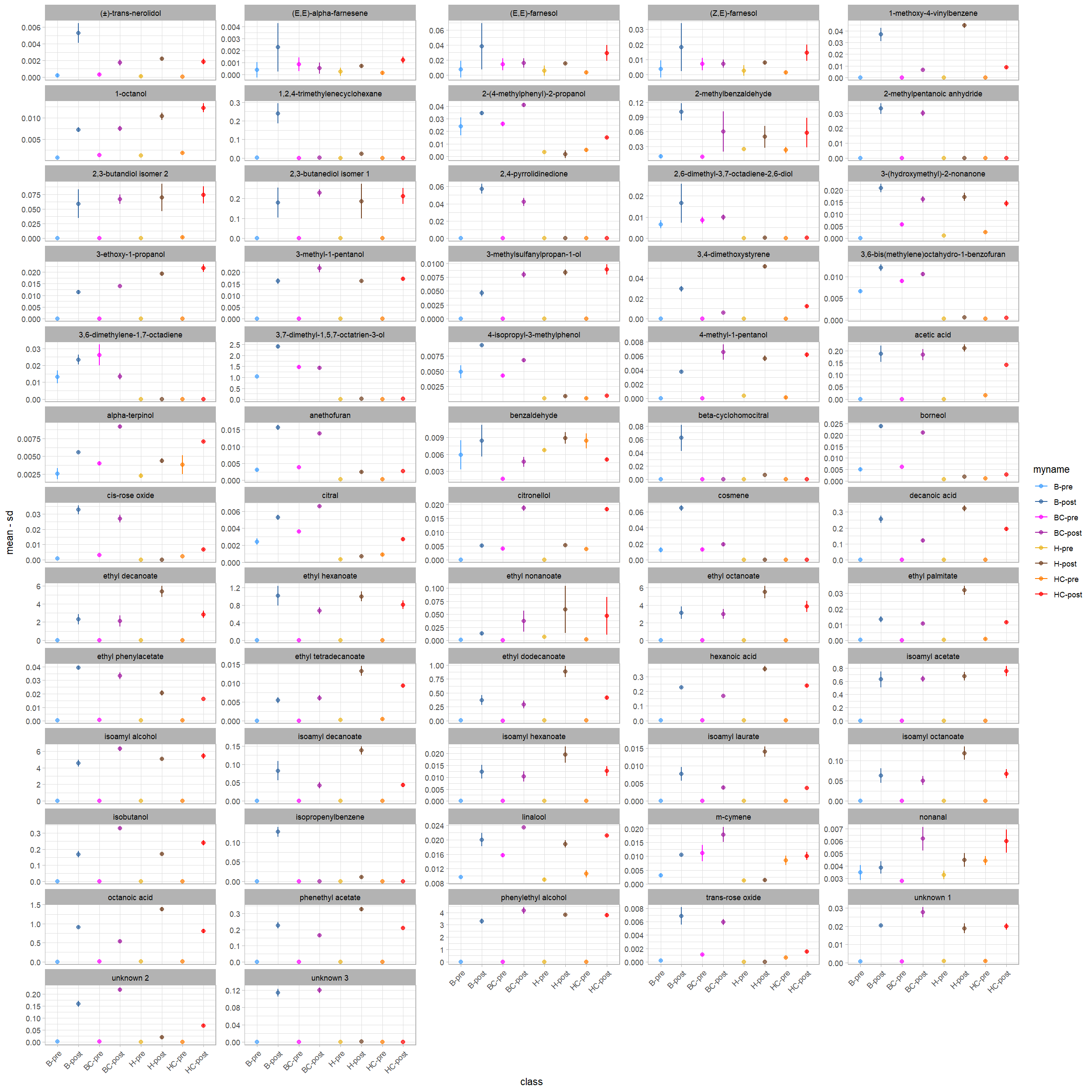

Supplement: Supplementary file 1 [file molecules-25-01818-s001.zip › molecules-771489 Figure S2.tiff]
